# Breaking Immunological Tolerance through OX40 (CD134)

**DOI:** 10.1100/tsw.2001.341

**Published:** 2001-11-06

**Authors:** Pratima Bansal-Pakala, Michael Croft

**Affiliations:** Division of Immunochemistry, La Jolla Institute for Allergy and Immunology, San Diego, Ca, USA

**Keywords:** tolerance, costimulation, autoimmunity, tumor, OX40

## Abstract

Immunological tolerance represents a mechanism by which cells of the host remain protected from the immune system. Breaking of immunological tolerance can result in a variety of autoimmune diseases such as rheumatoid arthritis, diabetes, and multiple sclerosis. The reasons for tolerance breaking down and autoimmune processes arising are largely unknown but of obvious interest for therapeutic intervention of these diseases. Although reversal of the tolerant state is generally unwanted, there are instances where this may be of benefit to the host. In particular, one way a cancerous cell escapes being targeted by the immune system is through tolerance mechanisms that in effect turn off the reactivity of T lymphocytes that can respond to tumor-associated peptides. Thus tolerance represents a major obstacle in developing effective immunotherapy against tumors. The molecules that are involved in regulating immunological tolerance are then of interest as they may be great targets for positively or negatively manipulating the tolerance process.

In contrast to the response to a foreign antigen, it has been proposed that either a lack of certain positive signals, or provision of certain negative signals, brought about by contacting membrane proteins, is required for T cell tolerance to be induced to self (non-foreign) antigens[1,2]. However, in general the nature of these signals has remained undefined. In the case of most tumor cells, for example, their poor immunogenicity is thought to be due to a failure to provide these costimulatory signals to tumor-specific T cells. Several prior reports have shown that reagents that enhance T cell stimulation at the time antigenic peptide is initially encountered can be effective in preventing tolerant T cells from developing. For example, it has been demonstrated that stimulatory antibodies targeting the CD40 molecule on antigen-presenting cells (APCs) can prevent the induction of T cell tolerance by enhancing the ability of these cells to present antigen. Another membrane molecule, termed CTLA-4, has additionally been shown to be a negative regulator of T cell function, and blocking CTLA-4 signaling has also been effective in preventing the induction of tolerance. However, it is likely that targeting these molecules is indirect and that other membrane proteins are the crucial effector molecules that may dictate whether T cell tolerance is operative.

A recent study published in *Nature Medicine*[3] has highlighted one molecule which may be critical to whether tolerance is maintained or is broken. Bansal-Pakala et al.[3] demonstrated that T cell tolerance can be prevented and, more significantly, reversed by providing signals through a novel inducible molecule expressed on the surface of T cells, termed OX40 (CD134). OX40 is a member of the TNF-R superfamily and is upregulated on both CD4 and CD8 T cells within 1–2 days of their encounter with antigenic peptide[4]. It binds to another inducible molecule termed OX40L predominantly expressed on APCs such as dendritic cells and B cells. Using several experimental models of tolerance in murine systems, the study by Bansal-Pakala et al. shows for the first time that an established state of tolerance in T cells can be reversed by providing signals through OX40. Treatment of previously tolerant animals with an agonistic anti-OX40 antibody promoted unresponsive antigen-specific T cells to greatly expand in numbers and to regain functionality. Significantly, providing OX40 signals only activated those T cells that could gain access to antigen, without any bystander activation of irrelevant T cells. These studies, therefore, have direct implications for generalized induction of autoimmunity and the use of OX40 targeting to allow only specific populations of T cells to be reversed from the tolerant state.

OX40 has previously been described as a molecule critical for host protection against foreign pathogens. Ligation of OX40 can increase clonal expansion of T cells and cytokine production *in vivo*[4,5], and it has been demonstrated that OX40 signals are required for long-term T cell survival and memory T cell development after immunization[6]. In particular, OX40 signals induce expression of the anti-apoptotic molecules, Bcl-2 and Bcl-xL, which function to protect the T cells from being susceptible to death-inducing stimuli[7]. The study by Bansal-Pakala et al.[3] now adds another important dimension to the function of OX40 and suggest it is also a molecule that, in some circumstances, may be detrimental to immune system homeostasis and may lead to autoimmunity if either it is aberrantly expressed or its ligand is aberrantly expressed. These observations highlight the possibility that OX40 may be involved in the generation of T cell responses that result in autoimmune disease, and that blocking OX40 signals may be a means of therapeutic intervention in a number of autoimmune diseases.

Conversely, the ability to reverse tolerance through OX40 also suggests that targeting this molecule may have tremendous benefit as an adjuvant to antigen-specific therapies aimed at augmenting immune function. Because OX40 only exerts its action when antigen is directly accessible to a T cell, the likelihood of inducing autoimmunity in a non-susceptible individual is very low. Experimental treatment with anti-OX40 antibody in rodents has already been shown to be effective in enhancing tumor rejection when given before significant growth has occurred[8]. The study by Bansal-Pakala now suggests that it may be feasible to enhance anti-tumor immunity by reversing tolerance of tumor-specific T cells, even after the tumor is well established. Future studies will no doubt explore the therapeutic potential of OX40 signals in generation of immune responses against established tumors.
